# Effect of Bufalin-PLGA Microspheres in the Alleviation of Neuropathic Pain *via* the CCI Model

**DOI:** 10.3389/fphar.2022.910885

**Published:** 2022-06-13

**Authors:** Lina Long, Wenwei Zhong, Liwei Guo, Jing Ji, Hong Nie

**Affiliations:** ^1^ Guangdong Province Key Laboratory of Pharmacodynamic Constituents of TCM and New Drugs Research, College of Pharmacy, Jinan University, Guangzhou, China; ^2^ International Cooperative Laboratory of Traditional Chinese Medicine Modernization and Innovative Drug Development of Chinese Ministry of Education (MOE), College of Pharmacy, Jinan University, Guangzhou, China; ^3^ School of Chinese Medicinal Resource, Guangdong Pharmaceutical University, Yunfu, China; ^4^ Guangzhou Nansha Information Technology Park Post-Doctoral Scientific Research Station, Guangzhou, China; ^5^ Guangzhou Bio-Green Biotechnology Co., Ltd., Guangzhou, China; ^6^ National Engineering Research Center of Pharmaceutical Processing Technology of Traditional Chinese Medicine and Drug Innovation, Guangzhou, China; ^7^ Guangzhou Dayuan Studio of Membrane Science and Technology for Traditional Chinese Medicine, Guangzhou, China

**Keywords:** neuropathic pain, CCI model, P2X7, bufalin, microspheres, dorsal root ganglia, sustained release, membrane emulsification

## Abstract

The treatment of neuropathic pain (NPP) is considered challenging, while the search for alternative medication is striving. NPP pathology is related with the expression of both the purinergic 2X7 (P2X7) receptor and the transient receptor potential vanilloid 1 receptor (TRPV1). Bufalin is a traditional Chinese medication derived from toad venom with pronounced antitumor, analgesic, and anti-inflammatory properties. However, poor solubility, rapid metabolism, and the knowledge gap on its pain alleviation mechanism have limited the clinical application of bufalin. Hence, the purpose of this study is to illustrate the NPP alleviation mechanism of bufalin *via* chronic constriction injury (CCI). To address the concern on fast metabolism, bufalin-PLGA microspheres (MS) were prepared *via* membrane emulsification to achieve prolonged pain-relieving effects. Western blot, real-time PCR, immunofluorescence, and molecular docking were employed to demonstrate the therapeutic action of bufalin on NPP. The results showed enhanced thermal withdrawal latency (TWL) and mechanical withdrawal threshold (MWT) after the administration of both bufalin and bufalin-PLGA MS in the CCI rats. Prolonged pain-relieving effects for up to 3 days with reduced dose frequency was achieved *via* bufalin-PLGA MS. In the CCI rats treated with bufalin-PLGA MS, the expression levels of protein and mRNA in TRPV1 and P2X7, both localized in the dorsal root ganglion (DRG), were reduced. Moreover, bufalin-PLGA MS effectively reduced the levels of IL-1β, IL-18, IL-6, and TNF-α in the CCI group. The results from molecular docking suggested a possible mechanism of NPP alleviation of bufalin through binding to P2X7 receptors directly. The administration of bufalin-PLGA MS prepared by membrane emulsification demonstrated promising applications for sustained effect on the alleviation of NPP.

## 1 Introduction

Pain is classified into two types, namely, chronic pain and acute pain. Chronic pain includes cancer-related pain, NPP, and inflammatory pain (Hu et al., 2014; Wang et al., 2015). Pain is recognized as a common disease, often accompanied by complex clinical features for proper diagnosis, particularly NPP-related symptoms. NPP occurs when the central nervous system or peripheral nervous system is damaged. The P2X purinoceptor is an ATP ligand–gated ion channel receptor. Damaged organs or tissues will trigger the release of ATP into the microenvironment of inflammation. By activating P2X receptors, ATP affects the equilibrium of the internal environment, thereby triggering an inflammatory response (Hamilton and McMahon, 2000; Jiang et al., 2013; Kobayashi et al., 2013). With the P2X purinoceptor binding to ATP, the ion channels in the cell membrane are activated to open up, causing an influx of sodium and calcium (Ca^2+^) ions and an outflow of potassium (K^+^) ions. In both glial cells and neurons, P2X expression is further upregulated, resulting in an increase in membrane potential activities. Consequently, the nervous system is sensitized, leading to the occurrence of pain (Kaczmarek-Hájek et al., 2012; Klein et al., 2012; Li et al., 2015). The P2X receptor are usually identified as 1–7 subtypes, in which P2X7R is included, mostly found in glia (Trang et al., 2012). Previous studies have verified that P2X7R is associated with NPP (Ursu et al., 2014).

TRPV1 (transient receptor potential vanilloid 1), as a membrane protein from the TRP channel family, has been shown to participate in a series of physiological processes and cellular activities, providing a potential therapeutic target for analgesic drugs. The expression of TRPV1 channels in DRG neurons for NPP varies in different animal models, while the specific mechanism of this phenomenon is still unclear. Studies have shown that the expression of TRPV1 channels in DRG neurons is promoted in the three models, namely, chronic constriction injury (CCI) model, diabetic neuropathic pain (DNP) model, and chemotherapeutic drug-related nerve injury model (Liu et al., 2018; Xing et al., 2019; Zhang et al., 2020). Glial cells have been reported to play a key role in affecting chronic pain and neuronal activities (Ji et al., 2013). In addition, the activated satellite glial cells (SGCs) participate in NPP, and neurons in DRG are capable of interacting with glial cells (Jarvis, 2010).

Traditional Chinese medicines have shown potential in treating many diseases for their anti-inflammatory, antiviral, immunoregulatory, analgesic, and antitumor effects. Chansu is toad venom mainly extracted from the skin of *B. melanostictus* and *Bufo gargarizans* (Krenn and Kopp, 1998). It has been used as a therapeutic substance for its apparent effects on many biological activities, such as antitumor properties, anti-inflammatory actions, and anesthetic effects (Chen and Kovaríková, 1967; Qi et al., 2011). Previous studies have suggested that bufalin, one of the most abundant substances obtained from Chansu, has been found to participate in various pharmacological processes, including cardiac protection, induction of apoptosis, inflammation reduction, and show analgesic properties (Wen et al., 2014; Kang et al., 2017; Song et al., 2017; Zhakeer et al., 2017). Owing to the special features of bufalin such as poor solubility in water, high toxicity, and rapid metabolism, its clinical use is severely limited (Gong et al., 2007).

A drug delivery system, particularly with microspheres involved, has opened up a new cross-disciplinary path for pharmaceutical research, which can be designed with unique properties to achieve higher water solubility, biocompatibility, reduced toxicity, and sustained release (Dahiya et al., 2007; Yin et al., 2012; Li et al., 2016). Conventionally, microspheres are prepared by mechanical stirring using high shear rotor-stator mixers and microhomogenizers. They are generally expected to produce high throughput while it is difficult to control the size of the microspheres, producing inhomogeneous microspheres from batch to batch (Håkansso, 2019). Membrane emulsification (ME) has been employed as an emerging technology for the preparation of colloidal emulsions for lipophilic drugs (Kobayashi et al., 2002). It is realized that the particle size and distribution can be tuned through process optimization and novel material design *via* ME technology. In contrast to high pressure homogenization, studies have demonstrated that ME technology produces smaller particle size with narrow distributions (Joseph and Bunjes, 2014). In this study, bufalin-PLGA (D, L-lactide-co-glycolide) microspheres were prepared by membrane emulsification technology using the laboratory-prepared PVDF membrane. PLGA is an FDA-approached biodegradable and biocompatible polymer (Abdelkader et al., 2017), which has been applied in many drug delivery systems to achieve sustained release. Microencapsulation of active pharmaceutical ingredients (APIs) could empower drug into a micro- or nanosized polymeric matrix for an enhanced solubility and bioactivity.

The aim of this work is to illustrate the NPP alleviation mechanism by bufalin and to verify bufalin-PLGA microspheres (bufalin-PLGA MS) prepared by membrane emulsification could extend the action on pain management. With the aid of network pharmacology, bufalin was selected as the interest of research in this study, which showed possible analgesic effect from preliminary results. By comparing the results from molecular docking coupled with *in vivo* experiments, that is, pain behavior, real-time PCR, Western blot, and immunofluorescence, the effects of bufalin on NPP alleviation were verified.

## 2 Materials and Methods

### 2.1 Materials

Poly(D, L-lactide-co-glycolide), ester terminated, inherent viscosity 0.55–0.75 dl/g, was obtained from LACTEL^®^ Absorbable Polymers (United States). Bufalin was purchased from Chengdu Alfa Biotechnology Co., Ltd., (China, CAS 465-21-4). Dichloromethane, polyvinyl alcohol (PVA), and Tween 80 were purchased from Shanghai Macklin Biochemical Co., Ltd. (China). Phosphate-buffered saline were obtained from Dulbecco A (PBS, OxoidTM, Thermo Fisher Scientific, Waltham, MA, United States), and acetic acid was purchased from Sinopharm Chemical Reagent Co., Ltd. (China). Dimethyl sulfoxide (DMSO) was purchased from Sigma-Aldrich (St. Louis, MO, United States). Morphine was provided by the Shanghai Institute of Materia Medica Chinese Academy of Sciences. Other reagents, solvents, and chemicals in this study were used as-purchased without further purification. Deionized water was used for the preparation of all aqueous solutions in this study.

### 2.2 Network Pharmacology

#### 2.2.1 Active Ingredient Screening and Target Prediction

Similar to earlier studies, the chemical compositions of Chansu were determined using a publicly available chemical database (http://bionet.ncpsb.org.cn/batman-tcm/) (Liu et al., 2021). All of the chemicals gathered from Chansu with their chemical structures were imported into the SwissADME database (http://www.swissadme.ch/). To screen the active components, the pharmacokinetics and drug similarities were anticipated. The aforementioned screening elements were entered into the SwissTargetPrediction database (http://www.swisstargetprediction.ch/). As a result, targets with a probability higher than 0.0 were recorded.

#### 2.2.2 Screening for Pain-Related Targets

The acquirement of pain-related targets was achieved by screening through the Online Mendelian Inheritance in Man (OMIM) database (https://omim.org/) and the GeneCards database (https://www.genecards.org/). Key terms, that is, “pain,” “analgesia,” and “neuropathic pain”, were used to conduct a thorough search for genes connected to pain. Genes in the Genecards database with correlation scores >1 were collected and integrated with those in the OMIM database genes, while overlaps from the results acquired before were removed. To screen putative targets for analgesic activity of Chansu, pain and active ingredient targets were intersected using R, and subsequently PPI network diagrams were constructed using the STRING 11.0 database (https://string-db.org/).

#### 2.2.3 Kyoto Encyclopedia of Genes and Genomes Analysis

In order to further screen the eight potential analgesic active ingredients obtained from the aforementioned experiments, molecular docking was performed between the core target proteins screened in the PPI and the main active ingredients screened by the network. In addition, many studies have shown that the P2X (Jacobson et al., 2020) and TRP (Jara-Oseguera et al., 2008) families have a clear role and promising potential in pain management, especially regarding NPP. Hence, receptors from these two families were included in the molecular docking section.

Chansu will be further investigated using KEGG by R and the Bioconductor data package, with a *p* < 0.05 threshold. The information gathered was expressed in bubble plots.

### 2.3 Preparation and Characterization of Formulations

**FIGURE 1:**
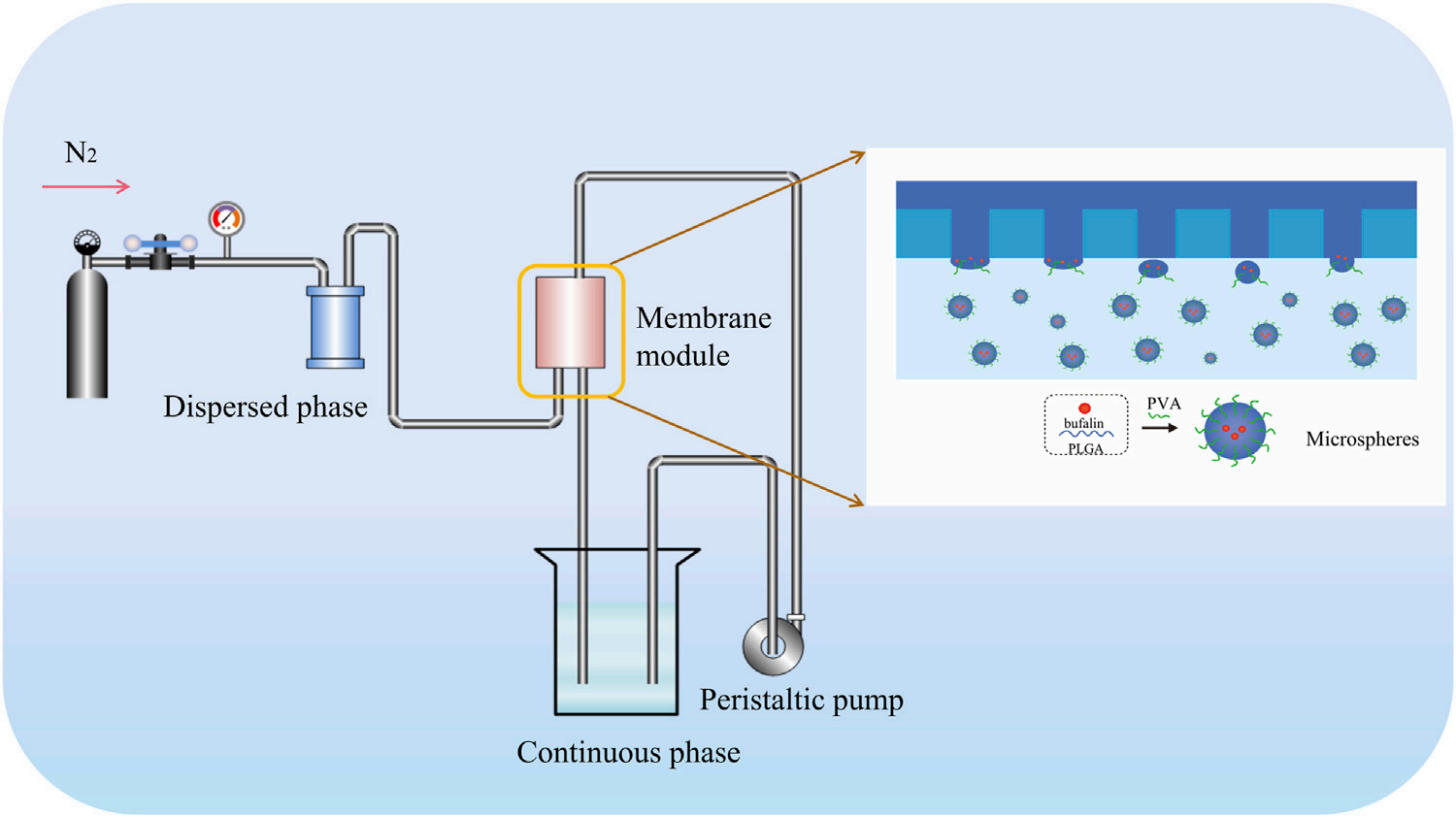
Schematic illustration of generating PLGA microspheres through PVDF membrane emulsification.

PVDF membranes have been fabricated in our lab. The preliminary results indicated that the membranes showed asymmetric hydrophilicity, which is generally considered favorable for the preparation of O/W emulsion (Zhong et al., 2022). A custom-built device (Figure 1) was used to conduct membrane emulsification. The oil phase, which contains bufalin and PLGA wall material, comes into contact with the hydrophobic side, during the ME process, whereas the aqueous phase, comprised of water and emulsifier, comes into contact with the hydrophilic side. With pressure applied on the oil phase, the oil phase permeated through the membrane, forming oil droplets on the membrane surface on the side of aqueous phase. The oil droplets detach from the membrane surface along with the circulated feed flow into the continuous phase, eventually forming the O/W emulsion for further processing. The entrapment rate and *in vitro* release rate were determined in previous experiments, which were used for the conversion of dosing consistent with that of free bufalin (Zhong et al., 2022). Characterization of bufalin-PLGA MS was performed by scanning electron microscopy (SEM, FEI Nova Nan SEM 450, United States). The size distribution of the as-prepared bufalin-PLGA MS in the SEM images were analyzed using ImageJ. A total of 200 particles at the magnification of ×5,000 were counted to determine the particle distribution.

### 2.4 *In Vivo* Evaluation of Analgesic Efficacy

#### 2.4.1 Acetic Acid–Induced Writhing Test

Healthy and clean KM rats (SPF, weight, 19–23 g) with equal number of females and males were purchased from Shanghai JSJ Laboratory Animal Company, and the use of animals followed the Animal Protection and Use Committee. All rats were kept in a sanitized room at 25°C ± 2°C and given adequate amount of water and feed. The animal laboratories were equipped with proper ventilation and air filtration systems. The rats were housed with a strict 12-h alternating light–dark cycle. License to use SYXK (Shanghai, 2020-0042).

The rats were initially subjected to an acetic acid twist experiment. The rats were placed in clear cages so that the twisting of the abdomen could be observed and calculated. The administration of bufalin of 0.125, 0.25, 0.5, and 1.0 mg/kg were set as the experimental groups, 5 mg/kg morphine as the positive group, and saline as the control group. Furthermore, 30 min after drug administration, 0.6% w/v acetic acid was injected. In addition, 5 min after the injection of 0.6% (w/v) acetic acid, the number of twists was counted and timed for a fixed period of 20 min. The mean number of torsions in each group and the percentage of torsion inhibition PIP were calculated using the following Eq. 1:

$$PIP=\frac{N_{c}-N_{a}}{N_{c}}\times 100\%,$$ (1)

where $N_{c}$ denotes the mean number of torsions in the control group and $N_{a}$ denotes the mean number of torsions in the administered group.

#### 2.4.2 Hot Plate Test

Healthy and clean female KM rats (SPF, weight, 19–23 g) were purchased from the Shanghai JSJ Laboratory Animal Company. Female rats were selected to eliminate possible intervention caused by the undesirable contact between the genitals of the male rats with the hot plate. The use of animals strictly followed the guidelines of the Animal Protection and Use Committee. All rats are housed in a clean room identical to abovementioned conditions. License to use SYXK (Shanghai, 2020-0042). The hot plate was maintained at 55°C ± 0.5°C (YSL-6B; Shanghai, China). Response latencies of rats were measured based on the time of action before the licking of hind paws or jumping after the placement on the heated surface. Bufalin doses of 0.5, 1.0, and 1.5 were set as experimental groups, with 5 mg/kg morphine serving as the positive control (Erichsen et al., 2005) and saline injection serving as the negative control. After the administration of drugs, hot plate latencies were assessed at a predetermined time span of 0, 30, 60, and 120 min.

#### 2.4.3 Chronic Constriction Injury Model Construction and Grouping

Healthy male Sprague Dawley (SD) rats (weight, 200–220 g) were purchased from Shanghai JSJ Laboratory Animal Company. The use of animals strictly followed the guidelines of Animal Protection and Use Committee. All animals are fed in a clean room identical to abovementioned conditions. Only male rats were chosen to eliminate possible intervention caused by cohousing both male and female rats for an extended period for the CCI model test. License to use SYXK (Shanghai, 2020-0042). The results of successful CCI construction and medication time are verified as shown in Figures 5A,B.

Bennett’s method was applied in this study (Bennett and Xie, 1998), with the procedures described below. Pentobarbital sodium was used to anesthetize the rat (40 mg/kg), followed by the exposure of the sciatic nerve *via* the separation of muscles, which was sealed up with 4–0 sutures. To finish up, four lightly ligated loops with 1 mm apart were properly knotted on the nerve. Nerves in the sham group (also known as control group) were shown to be exposed without knotting. The remaining steps were followed in a similar manner as the model group. Significant decrease in the mechanical withdrawal threshold (MWT) and the thermal withdrawal latency (TWL) were observed compared to the control group, indicating successful model construction.

To examine the effects of bufalin and bufalin-PLGA MS on NPP, rats were randomly divided into nine groups as follows: control group (sham group), CCI model group, CCI model + morphine group, CCI model +0.5 mg/kg bufalin group, CCI model +1.0 mg/kg bufalin group, CCI model +1.5 mg/kg bufalin group, CCI model +0.5 mg/kg bufalin-PLGA MS group, CCI model +1.0 mg/kg bufalin-PLGA MS group, and CCI model +1.5 mg/kg bufalin-PLGA MS group. Bufalin was dissolved in DMSO prior to administration for all tests in this study.

The administration started 10 days after the construction of the CCI model for the group of CCI + bufalin at a frequency of once per day for 7 days. The MWT and TWL were measured on the first day of bufalin administration at the predetermined time of 0.5, 1, 2, 4, 6, and 12 h. Similarly, MWT and TWL were measured at day 3, 5, and 7 at 0.5 h after administration of bufalin. Meanwhile, the same dose of saline was given to the control group, and the CCI model + morphine group was given 5 mg/kg morphine for 1 week. The administration of bufalin-PLGA MS was conducted in a similar manner as described before. However, the administration was only injected intraperitoneally once a week.

#### 2.4.4 Behavior Tests

MWT determination was conducted as the following similar to previous study (Jia et al., 2018). An electromechanical pain meter (BIO-EVF4, France) was used to test the MWT. The TWL measurement was conducted as the following procedures (Deng et al., 2018). The TWL was detected by a thermal radiation simulator (BME-390, United States). Each rat was measured five times, at 5-min intervals for each measurement. A blinded design was used for all experiments.

#### 2.4.5 Quantitative Real-Time PCR

The expression of biomarker mRNAs was measured by PCR following a similar manner as previous study (Yuan et al., 2018). Total RNA was prepared by using the TRIzol method, and the corresponding cDNAs were produced by using the PrimeScript™ RT reagent kit (Perfect Real-Time) (Takara, Japan). Reactions were performed in the Applied Biosystems 7300 Real-Time PCR System. To determine relative quantification, the 2^−ΔΔCT^ method was used.

The following primers were designed with Primer Express 3.0 software (Applied Biosystems):

For the P2X7 receptor (forward: 5′-GAC​AAA​CAA​AGT​CAC​CCG​GAT-3′, reverse: 5′-CGC​TCA​CCA​AAG​CAA​AGC​TAA​T-3′); TRPV1 receptor (forward: 5′-CCG​GCT​TTT​TGG​GAA​GGG​T-3′, reverse: 5′-GAG​ACA​GGT​AGG​TCC​ATC​CAC-3′); IL-1β (forward: 5′-GCA​ACT​GTT​CCT​GAA​CTC​AAC​T-3′, reverse: 5′-ATC​TTT​TGG​GGT​CCG​TCA​ACT-3′); IL-6 (forward: 5′-TCT​ATA​CCA​CTT​CAC​AAG​TCG​GA-3′, reverse: 5′-GAA​TTG​CCA​TTG​CAC​AAC​TCT​TT -3′); IL-18 (forward: 5′-GAC​TCT​TGC​GTC​AAC​TTC​AAG​G-3′, reverse: 5′-CAG​GCT​GTC​TTT​TGT​CAA​CGA-3′); TNF-α (forward: 5′-CCT​GTA​GCC​CAC​GTC​GTA​G-3′, reverse: 5′-GGG​AGT​AGA​CAA​GGT​ACA​ACC​C-3′); and GAPDH (forward: 5′-AGG​TCG​GTG​TGA​ACG​GAT​TTG -3′, reverse: 5′-GGG​GTC​GTT​GAT​GGC​AAC​A-3′).

#### 2.4.6 Immunofluorescence Staining

Immunofluorescence staining was used to detect the TRPV1 and P2X7 receptors expression in the DRGs. A sample of DRGs was fixed by 4% paraformaldehyde. The fixed tissue was rinsed with PBS solution and blocked by goat serum, prior to its incubation with anti-P2X7 (Affinity Biosciences, OH, United States) and anti-TRPV1 (Affinity Biosciences, OH, United States) overnight at 4°C. The DRGs slides were incubated with the goat anti-mouse fluorescent secondary antibody against isothiocyanate (FITC) (Affinity Biosciences, OH, United States) for 50 min and stained for 10 min by 4′, 6-diamidino-2-phenylindole (DAPI) (Beijing Solbio Technology Co., Ltd.) upon the treatment of PBS. The DRGs slides were finally sealed with an anti-fluorescence attenuator. A fluorescence microscope (Olympus, Tokyo, Japan) was employed to obtain imaging on the stained DRGs slides. The experimental procedure can be referred to the protocols mentioned in Ge et al. (2019).

#### 2.4.7 Western Blot Analysis

The protein extraction process in this study can be referred to the existing study of Yi et al. (2018). Protein weighing 20–30 µg were electrophoresed on SDS polyacrylamide gels. We then transferred the proteins to membranes, which were blocked with skimmed milk (5%) for 2 h at room temperature. Incubation of primary antibodies with the proteins was stored overnight at 4°C as follows: anti-P2X7 (Affinity Biosciences, OH, United States), anti-TRPV1 (Affinity Biosciences, OH, United States), anti-IL-18 (Affinity Biosciences, OH, United States), anti-TNF-α (Affinity Biosciences, OH, United States), anti-IL-6 (Affinity Biosciences, OH, United States), anti-IL-1β (Affinity Biosciences, OH, United States), or anti-GAPDH (Affinity Biosciences, OH, United States).

The membranes were rinsed with TBST for 10 min for triplicates and incubated for 2 h with the secondary antibodies (Boster Biological Technology Co. Ltd.). ECL chemiluminescence detection and development with a gel imaging system were performed finally. Results analysis for this section was realized *via* ImageJ.

### 2.5 Molecular Docking

The core network pharmacological targets CXCL8, MAPK1, PIK3CA, STAT3, CASR, and P2X family proteins P2X2, P2X3, P2X4, P2X7, and TRP family proteins TRPA1, TRPM8, TRPV1, TRPV2, TRPV3, and TRPV4 receptors sequences were obtained from http://www.UniProt.org/. Through the use of PyMOL, we removed small molecule ligands, dehydrated, and hydrogenated receptors. The bufalin was obtained from https://pubchem.ncbi.nlm.nih.gov/ and followed by the conversion to pdb format for further processing. Finally, auto dock vina 1.1.2 was used to perform molecular docking (Wang et al., 2021). To further analyze the protein–ligand interactions, a 2-dimensional picture of the active sites of the P2X7 receptor with bufalin was analyzed using LigPlot (Chamoli et al., 2016).

### 2.6 Statistical Analysis

SPSS 22.0 (IBM, United States) was used to conduct statistical analysis. We expressed all data as mean ± standard error (mean ± SEM). Data collected from both experimental and control groups was compared using the one-way ANOVA analysis followed by Fisher’s Least Significant Difference (LSD) procedure. The statistical significance was chosen at a confidence level of *p* < 0.05. GraphPad Prism 5 software was used for graphical analysis.

## 3 Results

### 3.1. Network Pharmacology of Chansu

**FIGURE 2:**
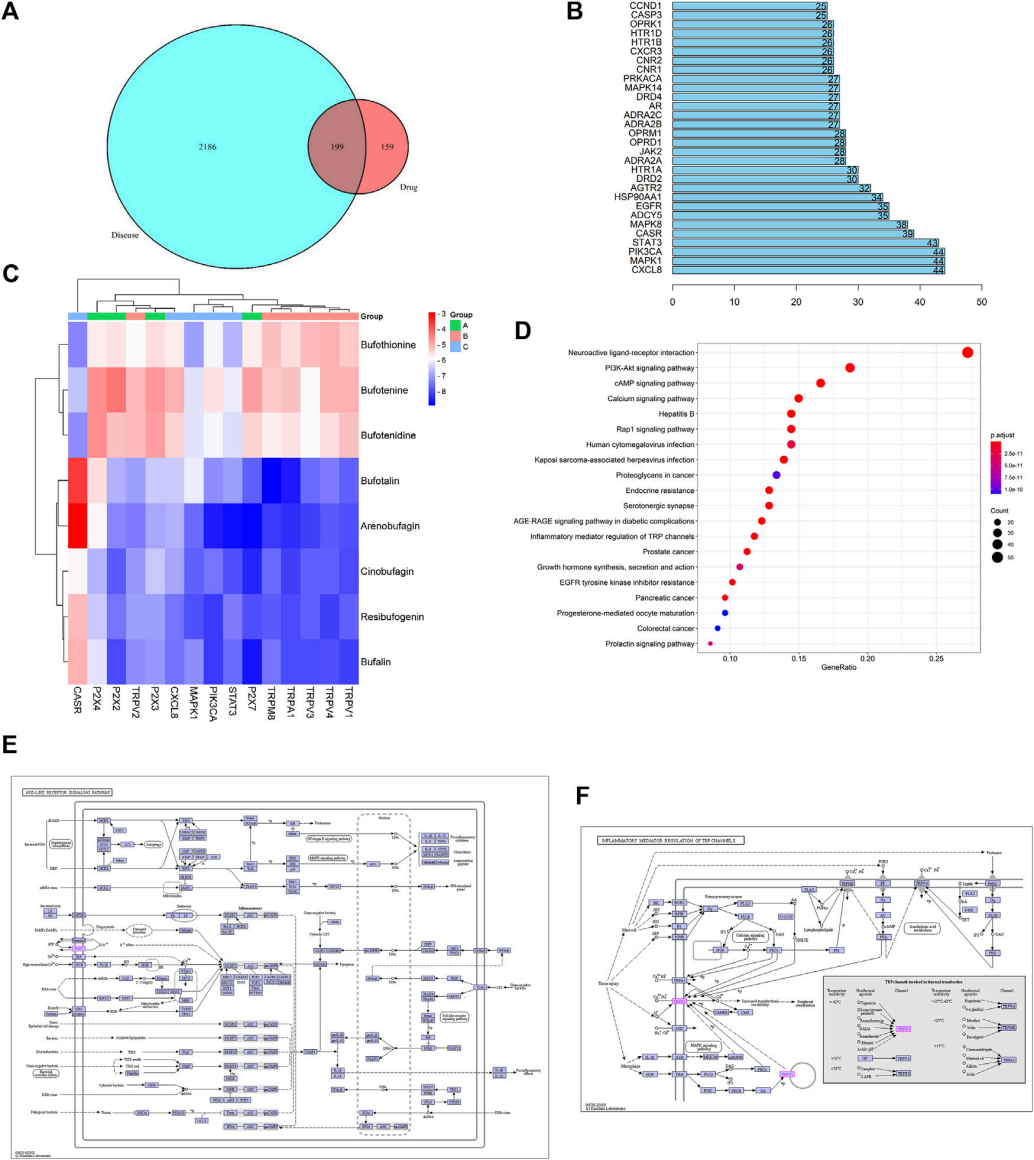
Network pharmacology prediction. **(A)** Wayne diagram of Chansu-pain targets. **(B)** Key targets of the Chansu protein interaction network for pain release. **(C)** Binding ability of the main active ingredient to the core target gene. **(D)** KEGG pathway enrichment analysis graph of Chansu. **(E)** Nod-like receptor signaling pathway. **(F)** TRP channels for inflammatory mediator regulation.

A total of 51 major chemical components of Chansu were screened and identified through SwissADME pharmacokinetic and pharmacodynamic predictions, including resibufogenin, cinobufagin, bufotalin and bufalin. From the analysis through Swiss Target Prediction database, it was found that eight compounds of Chansu are associated with 358 genes of target (Table 1). On the other hand, Genecards database suggested that a total of 11,676 genes could be related to pain. OMIM database also provided information on 18 genes that could be possibly linked to pain. Genes with a correlation score greater than 1 were selected while the overlapping genes from the Genecards database and OMIM database were excluded. As a result, 2,385 targets were obtained associated with pain-related sensation while a number of 199 pain-related targets were identified *via* the Venny analysis shown in Figure 2A.

**TABLE 1:** Main active ingredients of Chansu.

Ingredients	Pharmacokinetics			Druglikeness		
	GI absorption	Lipinski	Ghose	Veber	Egan	Muegge
Resibufogenin	High	Yes	Yes	Yes	Yes	Yes
Arenobufagin	High	Yes	No	Yes	Yes	Yes
Cinobufagin	High	Yes	Yes	Yes	Yes	Yes
Bufalin	High	Yes	Yes	Yes	No	No
Bufotalin	High	Yes	Yes	Yes	Yes	No
Bufotenidine	High	Yes	No	Yes	Yes	Yes
Bufotenine	High	Yes	No	Yes	Yes	Yes
Bufothionine	High	Yes	Yes	Yes	No	No

The binding capacity of these major active ingredients was predicted by molecular docking with the core network pharmacological targets CXCL8, MAPK1, PIK3CA, STAT3, CASR, and P2X family proteins P2X2, P2X3, P2X4, P2X7, and TRP family proteins TRPA1, TRPM8, TRPV1, TRPV2, TRPV3, and TRPV4 (Figures 2B,C). The eight main active ingredients were docked to 15 target proteins. The results indicated that bufalin, arenobufagin, resibufogenin, bufotalin, and Cinobufagin all showed strong binding capacities to the abovementioned 15 targets, with bufalin demonstrating the most enhanced binding capacity. Moreover, among the eight compounds mentioned above, bufalin showed the most enhanced analgesic effects as compared to various components in Chansu while little literature is readily available on its pain-relieving mechanism to date (Wen et al., 2014; Rong et al., 2014).

The potential analgesic pathways of Chansu were found by KEGG analysis, where the 20 pathways associated with pain are listed in Figure 2D. Among these pathways, Nod-like recepter signaling pathway (Figure 2E), PI3K-Akt signaling, and inflammatory mediator regulation of TRP channels (Figure 2F) were closely related to pain. In addition, to address the known concerns associated with bufalin such as toxicity, poor solubility in water and limited half-life, we suggested the preparation of prepared bufalin-PLGA microspheres (bufalin-PLGA MS) by membrane emulsification (ME) might be a feasible solution.

### 3.2 Preparation and Characterization of Bufalin-PLGA MS

**FIGURE 3:**
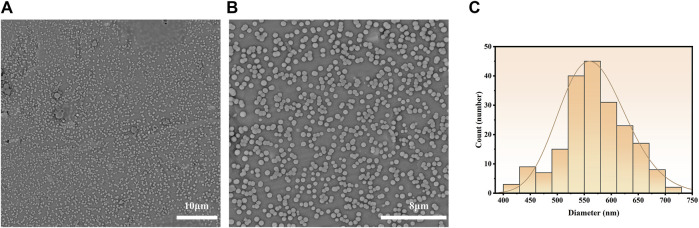
Particle morphology of bufalin-PLGA MS in SEM images and size distribution. **(A)** ×5,000 in SEM images. **(B)** ×10,000 in SEM images. **(C)** Size distribution.

The emulsions were dried on an aluminum foil and proceeded for SEM analysis. The as-prepared bufalin-PLGA MS exhibited spherical shapes and a particle size of 570 ± 60 nm, as shown in Figure 3. The encapsulation rate and *in vitro* release of microparticles were determined in preliminary experiments, with the entrapment efficiency of bufalin at a value of 85.10% and a loading rate of 3.84%. The *in vitro* cumulative release rate at 48 h was measured at 26.2% (Zhong et al., 2022).

**FIGURE 4:**
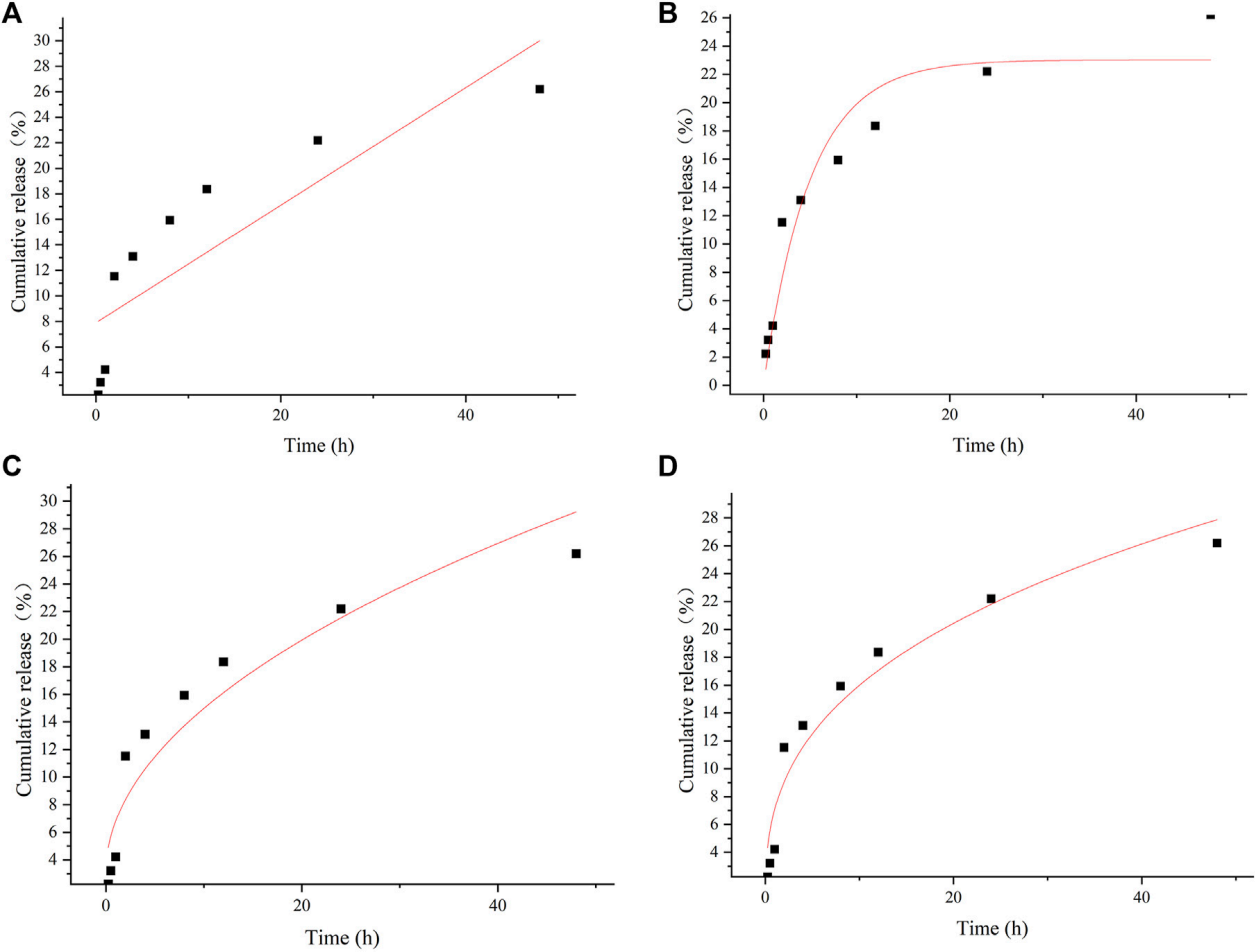
*In vitro* release curves for different model fits of **(A)** zero-order release, **(B)** first-order release, **(C)** Higuch, **(D)** Riger–Peppas.

Correlation coefficient (R^2^) of the data obtained from the pharmacokinetic analysis were evaluated. For selecting the most appropriate model to describe the sustained release profile of bufalin, the R^2^ value was used (Table 2). Zero-order, first-order, Higuchi and Ritger–Peppas model were used to fit the experimentally determined cumulative release respectively shown in Figure 4. The *in vitro* release simulation showed that the results were consistent with the Riger–Peppas model with the highest correlation coefficient R^2^. Fick’s diffusion can be assumed as the governing mechanism of the bufalin-PLGA MS in this study as reported previously responsible for controlling the drug release of Riger–Peppas model (Phaechamud et al., 2019).

**TABLE 2:** Kinetics of bufalin-PLGA MS release according to different kinetic models.

Release model	Regression equation	R^2^
Zero-order release	Q = 0.46t + 7.89	0.686
First-order release	Q = 23.01 (1-e^−0.2t^)	0.920
Higuch	Q = 3.78t^1/2^ + 3.02	0.892
Riger–peppas	Q = 7.07t^0.35^	0.937

### 3.3 Acetic Acid–Induced Writhing Test and the Hot Plate Test

**FIGURE 5:**
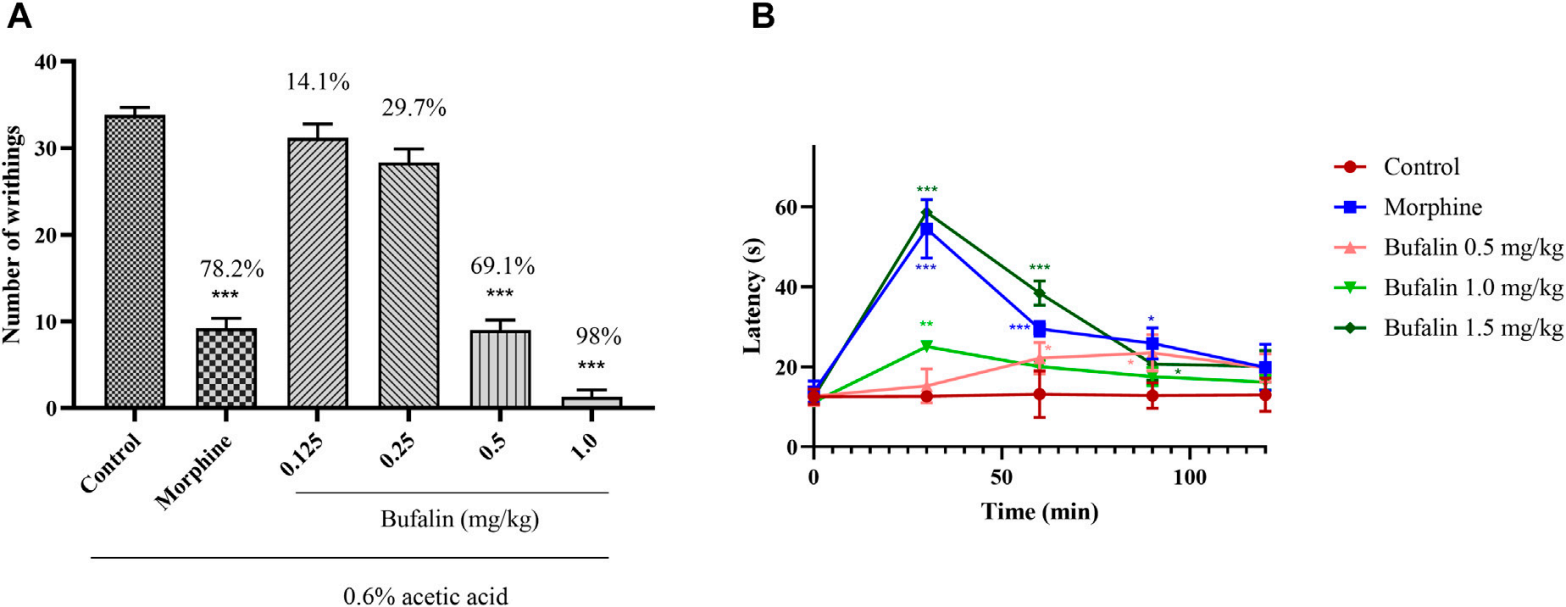
Results of acetic acid twist and hot plate. **(A)** Number of abdominal writhing movements for the rats administrated with bufalin at dosing of 0.125, 0.25, 0.5, 1.0, and 5 mg/kg morphine 30 min prior to the acetic acid injection. **(B)** Before the start of the hot plate experiment, 0.5, 1.0, and 1.5 mg/kg bufalin and 5 mg/kg morphine were administered, and the heat latency of the rats was measured at 0, 30, 60, 90, and 120 min after administration. Data represent mean ± SEM, *n* = 10. *p < 0.05, **p < 0.01, and ***p < 0.001 compared to the control group.

The two classical pain models of the acetic acid–induced writhing and hot plate were employed for initial assessment on the feasibility of bufalin for pain management. Both tests were generally applied for a preliminary screening of potential drugs that might be used for chronic pain management (Falade et al., 2019; Roy et al., 2019). Preliminary results on the abdominal writhing and the analgesic activity of bufalin in the rats after acetic acid injection was illustrated in Figure 5A. The group treated with the positive drug morphine of 5 mg/kg showed a remarkable reduction in twisting number (78.2%) (*p* < 0.001). No pain relief was observed in animals treated with bufalin of 0.125 and 0.25 mg/kg. In comparison with the control group, 0.5 mg/kg bufalin and 1.0 mg/kg bufalin were both able to inhibit nociceptive activity by 69.1% and 98% (*p* < 0.001), respectively.

It is important to mention that preliminary experiment showed that a dosing over 2.0 mg/kg could cause death to the rats and hence higher dosing was not considered for this study. The acetic acid–induced writhing test demonstrated that the bufalin does of 0.125 and 0.25 mg/kg is not sufficient to show analgesic effect. Hence the two low doses were excluded for further analysis *via* the hot plate test. In Figure 5B, the response latencies after administration of 5 mg/kg morphine and various doses of bufalin were recorded at 0, 30, 60, and 120 min. The results demonstrated that morphine increased the response latency at 30, 60, and 90 min (*p* < 0.05 or *p* < 0.001). Animals in the 0.5 mg/kg bufalin showed increased response latencies at 60 and 90 min compared to the control group (*p* < 0.05). The treatment of bufalin at 1.0 mg/kg increased the response latencies at 30 min (*p* < 0.01), while it failed to increase the reaction latencies at 60, 90, 120 min. The reaction latencies surged substantially in the group administrated with bufalin of 1.5 mg/kg at 30, 60, and 90 min (*p* < 0.05 or *p* < 0.001). The analgesic effect of all doses diminished after 120 min, suggesting that while bufalin has decent analgesic effects, it is rapidly metabolized in the body and does not provide a sustained pain management solution.

### 3.4 Chronic Constriction Injury Model Experiment

#### 3.4.1 Effect of Free Bufalin and Bufalin-PLGA MS in Chronic Constriction Injury Rats

**FIGURE 6:**
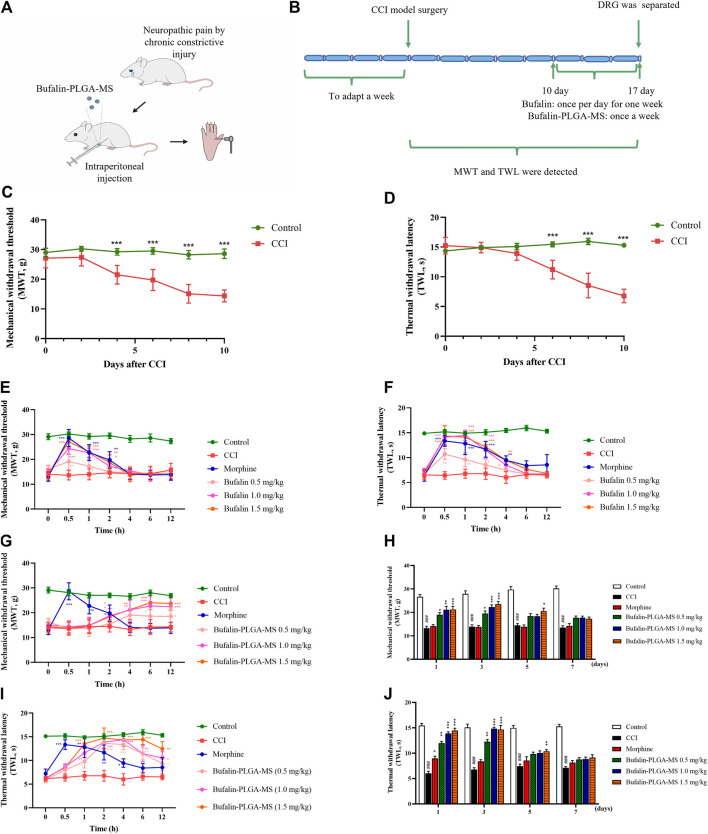
Results of pain behavior over time. **(A,B)** Schematic illustration of the model construction. **(C,D)** Model was successfully constructed 10 days after the CCI surgery. **(E)** MWT and **(F)** TWL in CCI rats were analyzed in different groups following administration of three different doses of free bufalin. **(G,H)** MWT and **(I,J)**TWL in CCI rats were analyzed in different groups following administration of three different doses of bufalin-PLGA MS. Data is expressed in the form of mean ± SEM, *n* = 8. *p < 0.05, **p < 0.01, and ***p < 0.001 compared to the CCI group.

The results of MWT and TWL are shown in Figure 6, where lower MWT and TWL usually indicate a higher pain sensitivity. Results for Figures 6C,D showed that 10 days after the construction of CCI model, the CCI group exhibited lower MWT and TWL sensitivities than the control group (*p* < 0.001), indicating the effective construction of CCI model.

The administration of 1.5 mg/kg bufalin exhibited an enhanced analgesic effect in comparison with the CCI group at 0.5, 1, and 2 h respectively in the MWT analysis (*p* < 0.05, *p* < 0.01 or *p* < 0.001) (Figure 6E), as well as at 0.5, 1, 2, and 4 h in the TWL analysis (*p* < 0.05, *p* < 0.01 or *p* < 0.001) (Figure 6F). In addition, the group with the administration of bufalin of 0.5 and 1.0 mg/kg showed analgesic effect to some extent, while the duration of action was shorter than 4 h (Figures 6E,F). The peak effect was found at 0.5 h after drug administration then the effect diminished rapidly.

On the other hand, all groups administrated with the bufalin-PLGA MS showed prolonged effects on NPP alleviation for up to 3 days (Figures 6H,J). It is important to point out that all dosing of bufalin-PLGA MS showed a remarkable and dose-dependent effect at predetermined time span of 2, 4, 6, 12, 24, and 72 h compared to the CCI group at the corresponding time in the MWT analysis (*p* < 0.05, *p* < 0.01 or *p* < 0.001) (Figures 6G,H) and TWL analysis (*p* < 0.05, *p* < 0.01 or *p* < 0.001) (Figures 6I,J). Groups administrated with bufalin-PLGA MS at 0.5 and 1.0 mg/kg showed a similar trend of analgesic effect, while the analgesic effect was less substantial than 1.5 mg/kg bufalin.

These results revealed that free bufalin and bufalin-PLGA MS could alleviate the MWT and TWL in CCI rats. It is worth noting that the as-prepared bufalin-PLGA MS could achieve sustained release and maintain decent analgesic effect for up to 3 days, while free bufalin exerted pain control for only 4 h.

#### 3.4.2 Effect of Bufalin-PLGA MS on Transient Receptor Potential Vanilloid 1 and Purinergic 2X7 mRNA Expression

**FIGURE 7:**
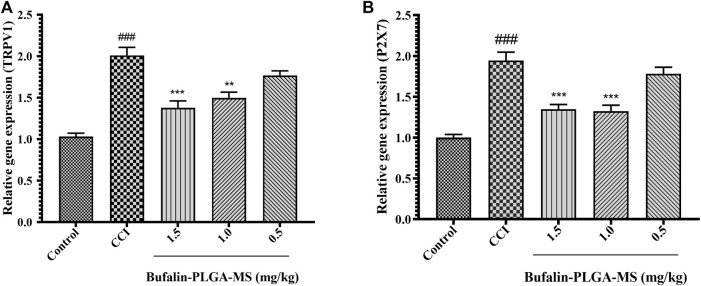
Bufalin-PLGA MS suppressed the TRPV1 and P2X7 mRNA expression in CCI rats. **(A)** mRNA levels of TRPV1 in every group. **(B)** mRNA levels of P2X7 in every group. Data is expressed in the form of mean ± SEM, *n* = 3. ###*p* < 0.001 compared to control group, *p < 0.05, **p < 0.01, and ***p < 0.001 compared to the CCI group.

Results of the PCR analysis indicated higher expression levels of the TRPV1 and P2X7 mRNA (*p* < 0.001) (Figure 7) were detected in the CCI group as compared to the control group. The expression levels of TRPV1 mRNA in the CCI + 1.0 mg/kg, 1.5 mg/kg groups were noticeably lower than CCI group (*p* < 0.01 or *p* < 0.001) (Figure 7A). On the other hand, insignificant difference was observed in the expression levels of TRPV1 mRNA between the CCI + 0.5 mg/kg group (*p* > 0.05) (Figure 7A) and the CCI group. The expression level of P2X7 mRNA was similar to that of TRPV1 (Figure 7B). The abovementioned results all suggested that the bufalin-PLGA MS might downregulate the mRNA expression of TRPV1 and P2X7 in the DRGs.

#### 3.4.3 Inhibition Effects of Bufalin-PLGA MS on the Expression of TRPV1 and Purinergic 2X7 Protein Levels in the DRG of CCI Rats

**FIGURE 8:**
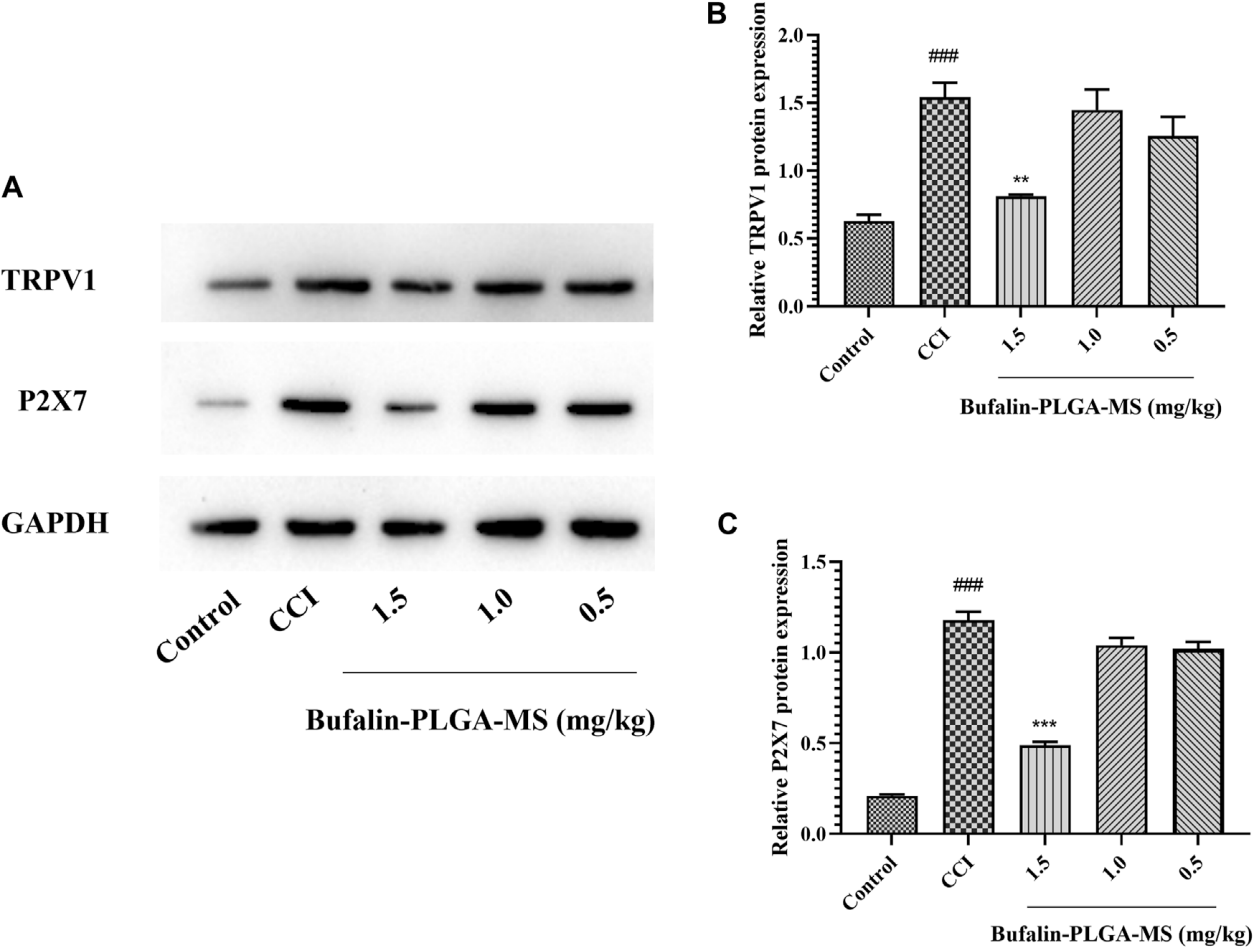
Bufalin-PLGA MS suppressed the protein expression of TRPV1 and P2X7 in CCI rats. **(A)** SDS-PAGE bands of TRPV1 and P2X7 protein in the DRG of different group rats. **(B)** Expression levels of TRPV1 protein in the DRG of different group rats. **(C)** Expression level of P2X7 protein in the DRG of different group rats. Data are expressed in the form of mean ± SEM, *n* = 3. ###*p* < 0.001 compared to the control group, *p < 0.05, **p < 0.01, and ***p < 0.001 compared to the CCI group.

The expression level of TRPV1 and P2X7 protein in DRG was measured *via* Western blot (Figure 8). Significantly higher levels of TRPV1 protein were detected in the CCI group compared to the control group (*p* < 0.001) (Figure 8B). The treatment of bufalin-PLGA MS of 1.5 mg/kg could inhibit the expression of TRPV1 protein in the DRG as compared to the CCI group without bufalin-PLGA MS treatment (*p* < 0.01) (Figure 8B). However, the expression of the TRPV1 protein in the DRG differed insignificantly in the groups of CCI + 0.5 or 1.0 mg/kg bufalin-PLGA MS and CCI group (*p* > 0.05) (Figure 8B). The expression level of P2X7 protein was similar to that of TRPV1 (Figure 8C).

#### 3.4.4 Immunofluorescence of Bufalin-PLGA MS on the of TRPV1 and P2X7

**FIGURE 9:**
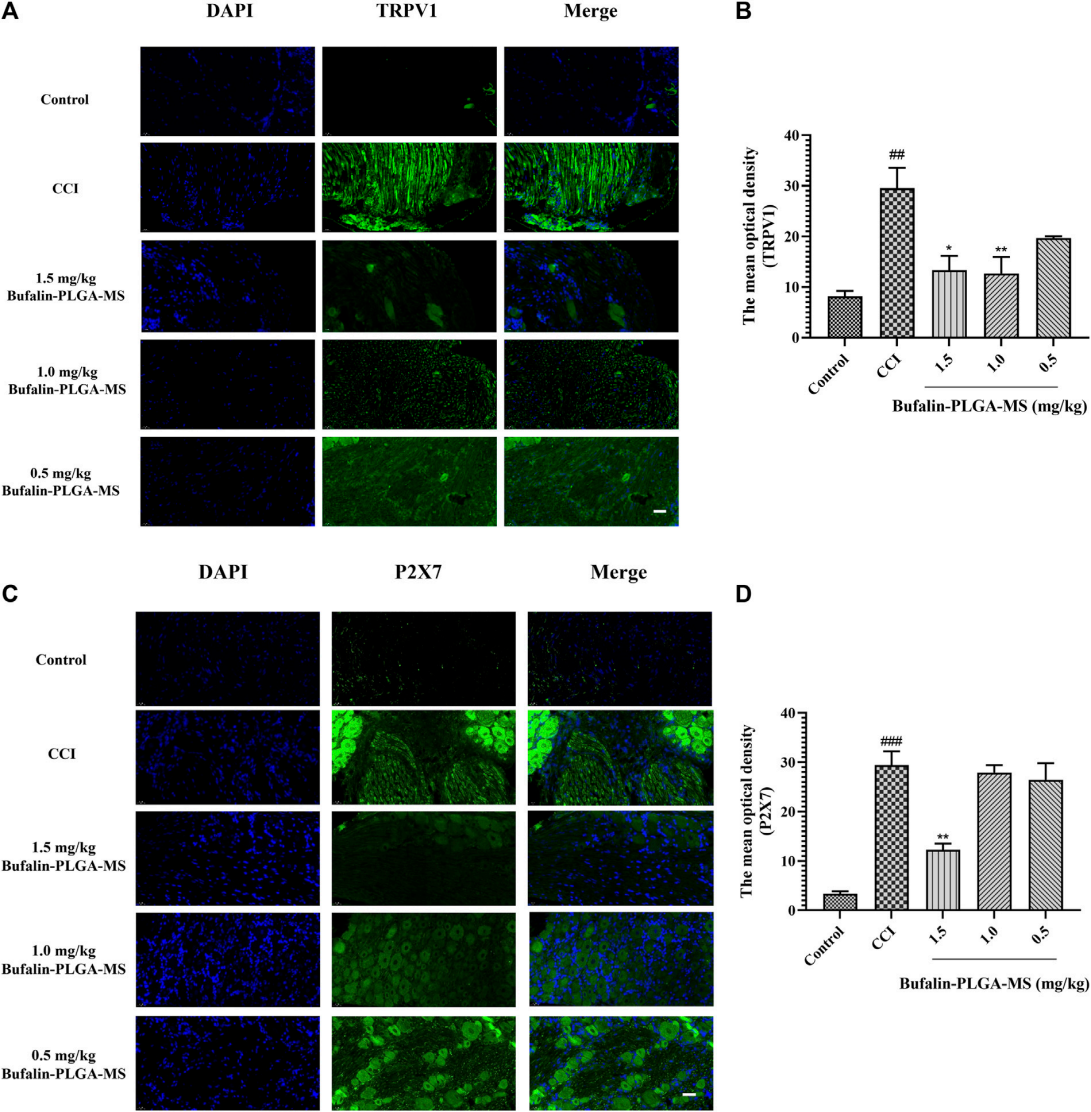
Expressions of TRPV1 and P2X7 were visualized and quantified *via* immunofluorescence staining. **(A)** Immunofluorescence detection of TRPV1 protein in the DRG; green and blue staining represent TRPV1 and DAPI, respectively. **(B)** Immunofluorescence detection of P2X7 protein in the DRG; green and blue staining represent P2X7 and DAPI, respectively. Data is expressed in the form of mean ± SEM, *n* = 3. ###*p* < 0.001 compared to the control group, *p < 0.05, **p < 0.01, and ***p < 0.001 compared to the CCI group. (Scale bar = 20 µm).

The expression levels of TRPV1 and P2X7 in the DRG were found to be substantially higher in the CCI group than the control group (*p* < 0.01, *p* < 0.001) *via* immunofluorescence staining shown in Figure 9. A lower level of TRPV1 expression was observed in the group treated with bufalin-PLGA MS of 1.5 and 1.0 mg/kg as opposed to the CCI group (*p* < 0.05, *p* < 0.01). Negligible change was observed between the CCI group and the 0.5 mg/kg bufalin-PLGA MS group. Therefore, it can be concluded that bufalin may inhibit the expression of the TRPV1 receptor in the DRG (Figures 9A,B).

P2X7 expression in the DRG was higher in the CCI group as compared to the control group (*p* < 0.001). Moreover, the expression of P2X7 was limited in the group of CCI + 1.5 mg/kg bufalin-PLGA MS, as opposed to the CCI group (*p* < 0.01). Insignificant difference was observed between the CCI group and the group administrated of bufalin-PLGA MS of 1.0 and 0.5 mg/kg. Hence, it is reasonable to conclude that bufalin may inhibit the upregulation of the P2X7 receptor expression in the DRG (Figures 9C,D).

#### 3.4.5 Molecular Docking

**FIGURE 10:**
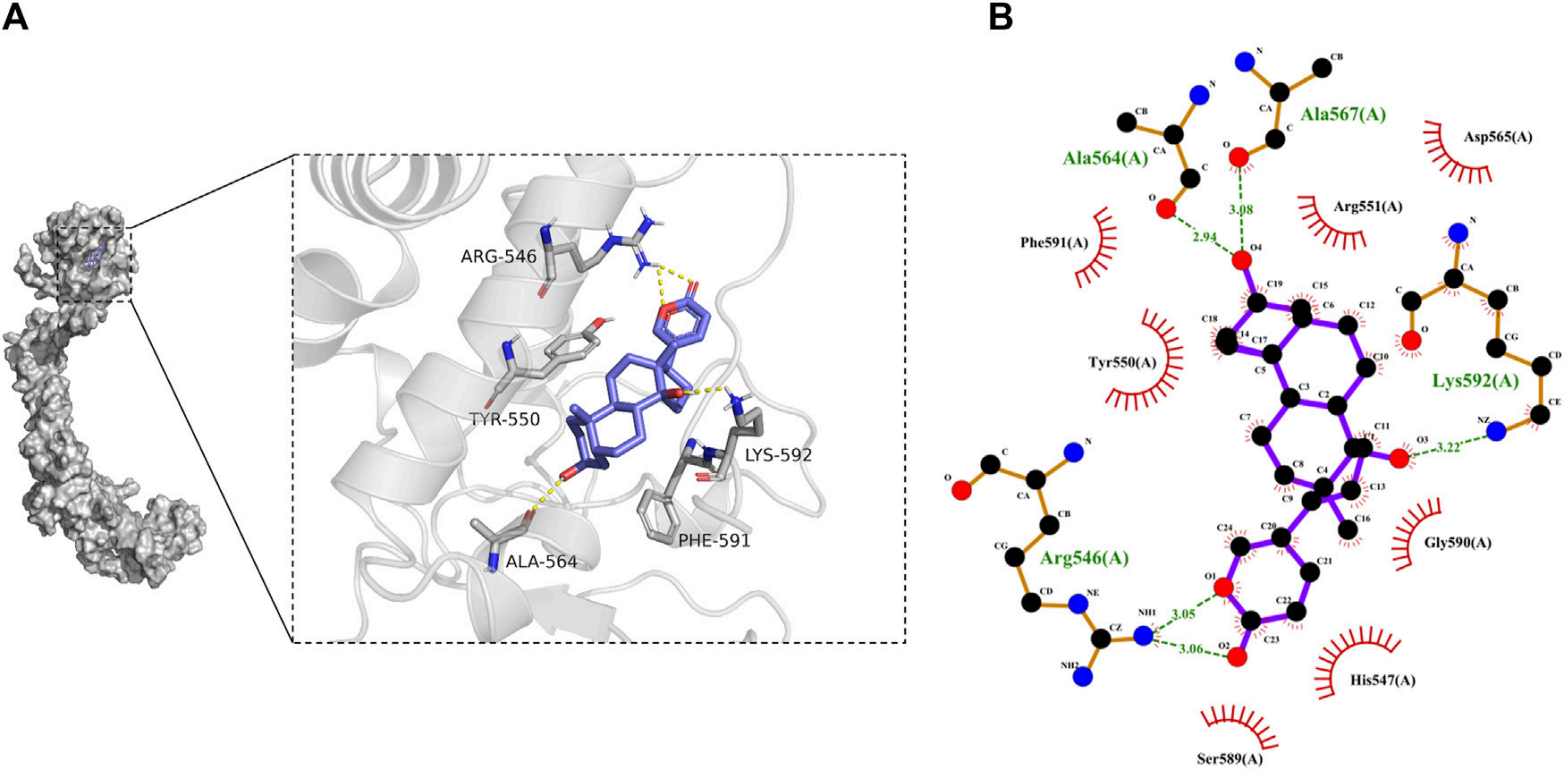
Results of molecular docking. **(A)** Molecular docking results showing the optimal binding site between bufalin and P2X7, where the yellow dashed line represents the hydrogen bond. **(B)** Diagram of the two-dimensional interactions involving several active amino acid residues of P2X7 with bufalin. Spoke arcs pointing towards the docked substrate represent hydrophobic interactions, atoms with spokes on the substrate indicate atoms involved in hydrophobic interactions, and hydrogen bonds are shown as green dashed lines.

The results from molecular docking indicated that binding affinity of bufalin to the TRPV1, P2X3, P2X4, and P2X7 receptors were estimated at values of −8.1, −7.1, −6.2, and −8.6 (kcal/mol), respectively. The binding ability of bufalin to the P2X7 receptor was the strongest among these receptors, by considering the absolute affinity value with >6 kcal/mol as the selected standard (Table 3). Moreover, bufalin can form three hydrogen bonds with the P2X7 receptor (Figure 10).

**TABLE 3:** Docking scores of P2X7 protein and bufalin (kcal/mol).

Mode/Rank	Affinity (kcal/mol)	Distance from rmsb* l.b	Best mode rmse u.b
1	−8.6	0.000	0.000
2	−8.3	3.516	5.986
3	−8.2	3.014	4.813
4	−8.0	2.096	3.918
5	−7.9	3.049	5.117
6	−7.9	3.570	7.534
7	−7.8	3.797	6.416
8	−7.7	2.918	5.069
9	−7.7	3.233	7.821

Explanation: The docking binding affinity was expressed in the unit of kcal/mol (energy). The specific description is as described in the literature (Yang et al., 2021).

As illustrated in Figure 10B, there are a number of amino acid residues such as PHE-591, TYR-550, ARG-551, ASP-565, GLY-590, HIS-547, and SER-589 forming a hydrophobic pocket, resulting in the encapsulation of the bufalin molecule. It might suggest that P2X7 can interact with bufalin *via* hydrophobic interaction. In addition, hydrogen bonds were found between bufalin and ALA-564, ALA-567, LYS-592 and ARG-546. The hydrogen bond lengths formed were estimated ranging from 2.94 to 3.22 Å, all exhibiting robust forces. The atom on the residue of LYS-592 in P2X7 and the atom on bufalin formed robust bonding with a length of 3.22 Å. The hydrogen bond length between the atom on the ALA-567 residue and the atom in bufalin was estimated at a value of 3.08 Å. In addition, LYS-592 and the ALA-567 amino acid residue could form the most stable hydrogen bond. The pain alleviation by bufalin can be explained by the hydrogen bonding and hydrophobic interactions between bufalin and P2X7. From the abovementioned results, we could speculate that NPP alleviation mechanism of bufalin involves the direct binding with the P2X7 receptor, which further induces the intervention with TRPV1 while the effects of bufalin on TRPV1 receptors are indirect.

#### 3.4.6 Effect of Bufalin-PLGA MS on the mRNA Levels of IL-18, TNF-α, IL-1β, and IL-6 in the DRGs of CCI Rats

**FIGURE 11:**
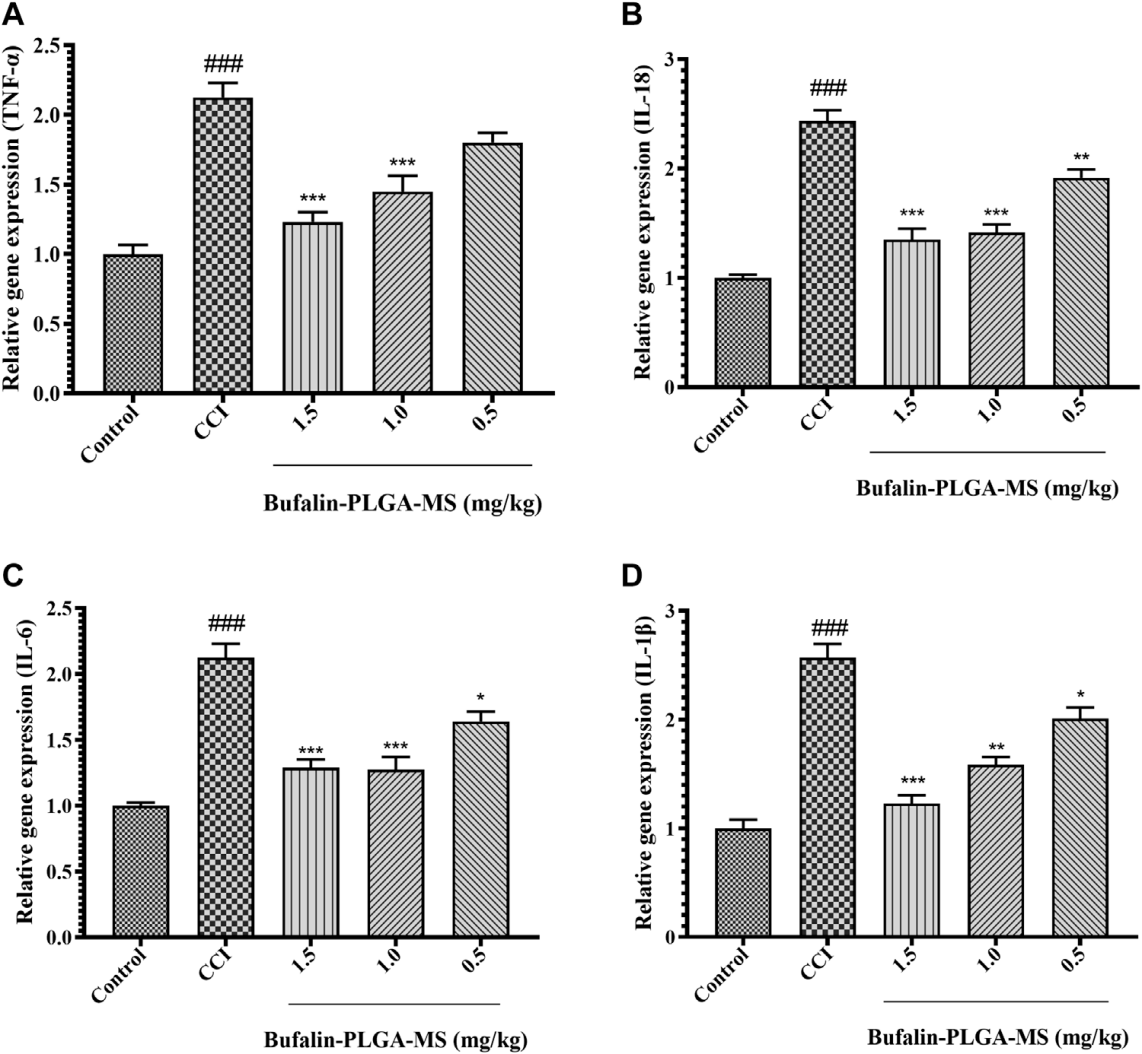
Effects of bufalin-PLGA-MS on the expression of the TNF-α, IL-18, IL-6, and IL-1β mRNA in the DRG of the CCI model. **(A)** The relative level of TNF-α mRNA expression. **(B)** The relative level of IL-18 mRNA expression. **(C)** The mRNA expression level of IL-6. **(D)** The mRNA expression level of IL-1β. Data is expressed in the form of mean ± SEM, *n* = 3. ### *p* < 0.001 compared to control group, * *p* < 0.05, ** *p* < 0.01 and *** *p* < 0.001 compared to CCI group.

Based on the data shown in Figure 11, it was confirmed that the level of gene expression of pro-inflammatory cytokines in the CCI group can be affected by the treatment of bufalin-PLGA MS. The mRNA expression levels of pro-inflammatory cytokines, that is, IL-18, IL-6, and IL-1β declined notably in the CCI +0.5, 1.0, and 1.5 mg/kg groups as opposed to the CCI group (*p* < 0.05, *p* < 0.01 or *p* < 0.001) (Figures 11B–D). The TNF-α gene expression in the CCI +1.0 and 1.5 mg/kg groups significantly reduced (*p* < 0.001). Insignificant difference was observed between the CCI +0.5 mg/kg and the CCI group (*p* > 0.05) (Figure 11A). The abovementioned results indicated that bufalin-PLGA MS may efficiently downregulate the transcription of pro-inflammatory genes, including IL-18, TNF-α, IL-1β, and IL-6 in the CCI modeled rats.

#### 3.4.7 Effect of Bufalin-PLGA MS on the Protein Levels of IL-18, TNF-α, IL-1β, and IL-6 in the DRGs of CCI Rats

**FIGURE 12:**
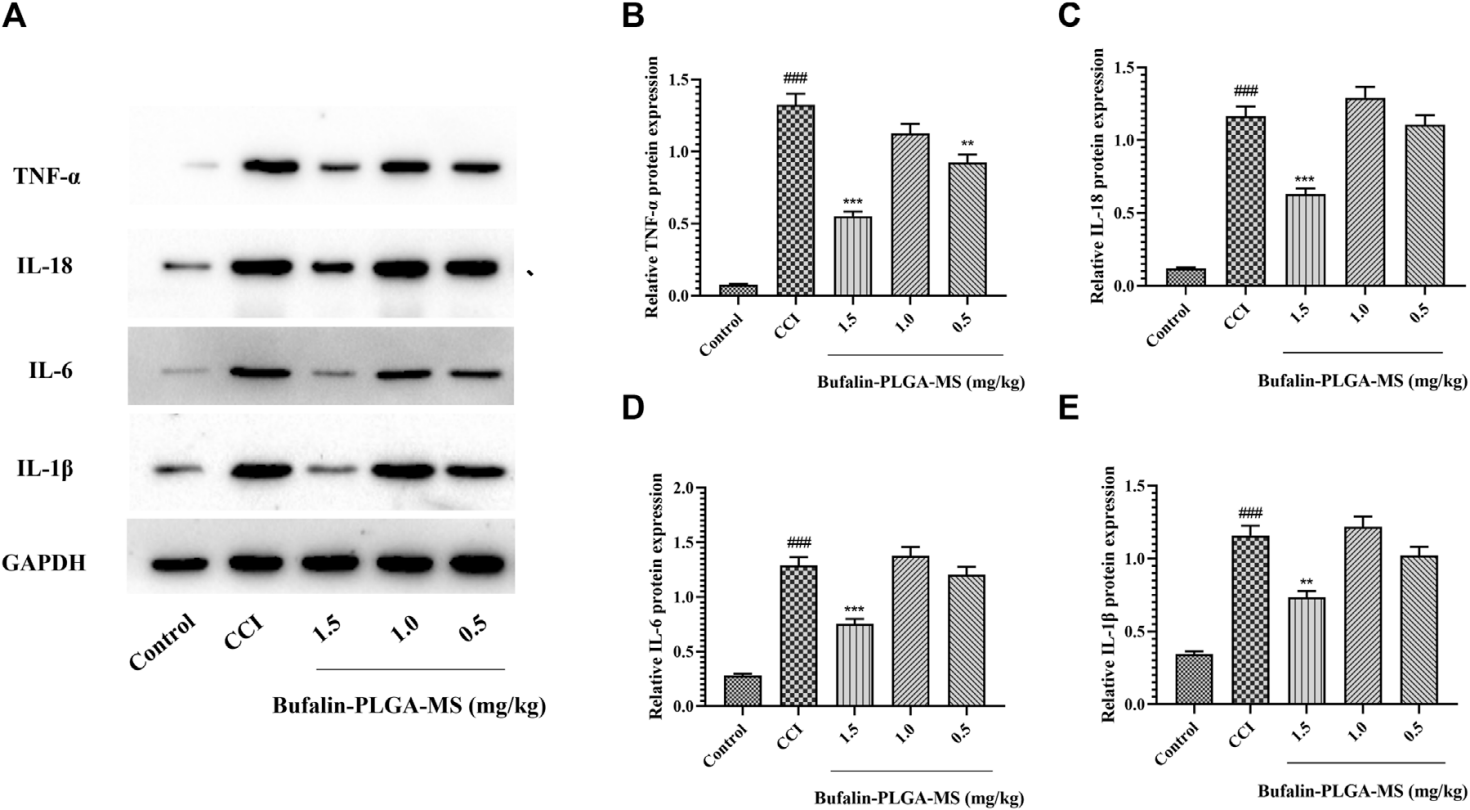
Protein levels of IL-18, TNF-α, IL-1β, and IL-6 in the DRG. **(A)** SDS-PAGE band of IL-18, TNF-α, IL-1β, and IL-6 in each group. **(B)** TNF-α protein expression in DRG. **(C)** IL-18 protein expression in DRG. **(D)** IL-6 protein expression in DRG. **(E)** IL-1β protein expression in DRG. Data are expressed in the form of mean ± SEM, *n* = 3. ### *p* < 0.001 compared to the control group, * *p* < 0.05, ** *p* < 0.01, and *** *p* < 0.001 compared to the CCI group.

A surge in cytokine expression is another key feature of SGCs activation. The initiation, development and maintenance of NPP are believed to be the consequence of the release of IL-18, TNF-α, IL-1β, IL-6 etc. from SGCs. The expression of TNF-α, IL-18, IL-6, and IL-1β proteins was higher in the CCI group than in the control group (*p* < 0.001) (Figure 12). The levels of IL-18, TNF-α, IL-1β, and IL-6 protein in DRG remarkedly decreased after the treatment of 1.5 mg/kg bufalin-PLGA MS as compared to the CCI group (*p* < 0.01 or *p* < 0.001) (Figure 12). Moreover, the group treated with 0.5 mg/kg bufalin-PLGA MS inhibited the expression levels of TNF-α protein in DRG as compared with the CCI group (*p* < 0.01) (Figure 12B).

## 4 Discussion

The physiological process of pain involves complex mechanism, usually associated with many possible causes. Chronic pain is one of the most challenging pathological states to manage, requiring a long-term treatment plan (Nazıroğlu et al., 2020). Often, NPP is caused by various neurological related diseases. Although the mechanism of NPP has been thoroughly discussed, the treatment for NPP still requires careful research and development. There are several types of drugs available to treat neuropathic pain, including NSAIDs, antidepressants, anticonvulsants, and opioids (Yi et al., 2018).

Eight potential analgesic components of Chansu, including resibufogenin, cinobufagin, bufotalin, and bufalin, were screened using network pharmacology, which were further verified to be associated with pain targets by molecular docking techniques. It was found that bufalin had the best binding ability among all, which can potentially be considered as a desirable candidate for analgesic purpose and pain management. Therefore, classical pain models were employed to confirm the analgesic effect and mechanism of bufalin.

The behavioral results in the acetic acid twist, hot plate and CCI model experiments showed that bufalin could alleviate pain, aligning with the results of virtual docking experiments. On the other hand, it is also worth noting that although bufalin demonstrated decent analgesic effect, the duration of action is often rapid, particularly with the peak effect observed at 0.5 h in our case. After 4 h, the effect diminished, indicating a more frequent dosing is needed to achieve a long-term analgesic effect. It implies that bufalin alone might not be appropriate for the chronic pain management, considering it as a persistent condition with a recurrent and prolonged course, which often requires a prolonged treatment by drugs with sustained effect. Hence drug design for the treatment of chronic pain and NPP should lie in the scope of discovering compounds with decent pain-relieving effects as well as the design of such drug delivery system with prolonged effects.

Process-optimized microspheres and nanoformulations can be used to achieve sustained or controlled drug release through drug diffusion and polymer dissolution once inside the body. Microencapsulated drugs are expected to degrade at a designated rate in the body, to achieve a reduced frequency of administration and to improve patient compliance (Kapoor et al., 2015). Conventional approaches to microsphere preparation include spraying and mechanical stirring, with concerns generally known as high energy consumption, inability to produce particles with uniformity and the lack of reproducibility of the product to fulfill the standard of GMP (Romanowsky et al., 2012; Abbaspourrad et al., 2013). Membrane emulsification is an emerging technology used in pharmaceutical industry for the microencapsulated APIs, usually operated at mild conditions. The prepared microspheres are expected to present uniform and controllable particle size, showing decent reproducibility.

The characterization of the bufalin-PLGA MS showed a uniform size of 570 nm in average, with a smooth surface. The *in vitro* release behavior of bufalin-PLGA MS was in accordance with the Ritger–Peppas model, with a cumulative release rate of 26.2% at 48 h. The analgesic effect of bufalin-PLGA MS was then carried in the *in vivo* experiment using a rat CCI model.

After the treatment of the CCI model rats with bufalin-PLGA MS, the MWT and TWL of the CCI model rats were significantly enhanced, indicating that the bufalin-PLGA MS was still feasible to alleviate the pain behavior of the CCI model rats. As previous results indicated in Figure 6, bufalin-PLGA MS showed a prolonged effect on the MWT and TWL than the CCI rats administrated with free bufalin. The analgesic effect was still apparent within 3 days after administration in the case of bufalin-PLGA MS administration. As previously mentioned, the effects of free bufalin peaked at 0.5 h in the groups with different dosing while it diminished after 4 h of administration. It has shown that membrane emulsification technology could prepare bufalin-PLGA MS with prolonged analgesic effects. However, the pain-relieving effect of the bufalin-PLGA MS was not obvious within 3 h after administration. It is important to mention that although it is not recommended to have a release rate exceeding certain amount as it will cause burst release (Kim et al., 2021), the design of drug release for analgesic purpose should consider an initial burst to create the immediacy of drug efficacy, allowing the drug diffusion to trigger faster onset of action.

In addition, it was noticed the existence of divergences between the *in vivo* efficacy and *in vitro* release. In the *in vitro* experiments, the cumulative release of the drug was less than 30% at 48 h, demonstrating decent sustained release. *In vivo* experiments showed that the pain alleviation effect of the drug did not exceed 5 days with a significant decrease in the pain-relieving effect after 3 days of administration. Firstly, the test of *in vitro* release is an approach to mimic the *in vivo* situation where most studies applied the *in vitro* release of microspheres as a useful indicator for the *in vivo* release. However, the *in vitro* release can only be emulated according to agitation, temperature and pH whereas the *in vivo* microenvironment is complex, and the *in vitro* release might not be representative for *in vivo* release (Siepmann et al., 2004; Otte et al., 2021). Moreover, the size of the microspheres prepared by membrane emulsification might be too narrow, causing rapid release at certain time. It has been hypothesized that at some point after administration, microspheres of similar size experience simultaneous dissolution to allow the drug to leak out and diffuse, resulting in a sudden release of the bufalin in this case (Li et al., 2019). Lastly, it is speculated that the size of the bufalin-PLGA MS prepared by membrane emulsification might not be large enough to provide analgesic effects for a longer duration. Previous studies have indicated that the release rate can be further prolonged by increasing the size of microspheres (Fu et al., 2005; Klose et al., 2006). Other factors such as drug load, can also affect the *in vivo* release of bufalin (Shi et al., 2003; Malavia et al., 2015). It is anticipated that the optimal analgesic effects of bufalin-PLGA MS can be achieved by selecting proper size distribution and dosing, which can be realized by membrane emulsification *via* process optimization and membrane design, for a desirable sustained release and reduced frequency of administration.

Finally, we have conducted a series of experiments to elucidate the mechanism of alleviation of NPP for bufalin-PLGA MS using PCR, Western blot, and immunofluorescence. Neurons have always been the main interest of research for pain treatment, but not until recently, some revealed that pain treatment may be resolved by targeting the drug on glial cells (Yang et al., 2021). P2X7 receptor is an important receptor in glial cells that influences the pathophysiological process of neuropathic pain. The TRP channel is a cation channel that is closely associated with various types of pain and plays an essential role in the maintenance of normal physiological functions in the body. Upon the construction of CCI model, the nervous system of the animal will be severely damaged. This will cause a substantial amount of ATP, histamine and capsaicin released in the system, further activating the P2X7 and TRPV1 receptors in the neighboring glial and neuron cells. The series of action will cause the additional release of ATP from the glial cell, and the amplification of the signals for ATP. The amplified signals will accelerate the overexpression of P2X7 and TRPV1, resulting in the release of pro-inflammatory cytokines, eventually leading to the sensation of pain. Hence, P2X7 and TRPV1 are usually overexpressed in the damaged nervous system, and the level of expression is positively proportional to the extent of damage experienced (Chessell et al., 2005; Ying et al., 2014).

In addition, the control of chronic pain is aided by neuroimmune responses. Several pro-inflammatory cytokines, including IL-1β, IL-6, IL-18, and TNF-α, have been implicated in the development of neuropathic pain. IL-1β indirectly activates TRPV1 by inducing the expression of COX-2 to induce various nociceptive sensations (Araldi et al., 2013). Increased expression of IL-6 is required for the pathogenesis of neuropathic pain, and the downregulation of this mediator has been shown effective in pain alleviation (Lee et al., 2002). IL-18 receptors are expressed in different cell types, including neurons and glial cells. Recent studies have shown the NPP alleviation mechanism by IL-18–mediated signaling pathways is through regulating the interaction between microglia and astrocytes (Guo et al., 2014). TNF-α is an important cytokine that plays a pivotal role in the pathological process of NPP, where the release of it is induced by P2X7 indirectly *via* exosomes, thereby inducing pain (Dai et al., 2020).

As a result of the experiments, bufalin-PLGA MS has been shown to inhibit the expression of TPRV1 and P2X7 in DRG, as well as to downregulate the pain-related inflammatory factors such as TNF-α, IL-1β, IL-18, and IL-6 in DRG, exerting an analgesic effect on CCI modeled rats. Coupled with the results from molecular docking, bufalin demonstrated enhanced binding affinity with P2X7 and formed three hydrogen bonds for a stable binding, while the binding affinity with TRPV1 is lower than that of P2X7. It is speculated that the NPP alleviation mechanism of bufalin can be explained by the direct interaction of bufalin with P2X7 receptor which further inhibiting TRPV1 indirectly.

## 5 Conclusion

Bufalin-PLGA MS with prolonged analgesic effects was successfully prepared by membrane emulsification. Bufalin and bufalin-PLGA MS both showed promising pain-relieving effects in the *in vivo* experiments using acetic acid twist assay, hot plate assay, and CCI model. Bufalin-PLGA MS could significantly prolong the duration of analgesia and reduce the number of administrations as a comparison to the free bufalin. The results revealed that NPP alleviation mechanism might through the direct interaction of bufalin on the P2X7 receptor, further inhibiting the expression of TRPV1 and regulating inflammatory factors such as IL-1β, TNF-α, IL-18, and IL-6. Future optimization of the bufalin-PLGA MS will include the design of size distribution and drug load to achieve analgesic effects with proper release profile lasting for a desirable time span.

## Data Availability

The datasets presented in this study can be found in online repositories. The names of the repository/repositories and accession number(s) can be found in the article. Further inquiries can be directed to the corresponding authors.
