# Supplementary figures and images for: Effect of Bufalin-PLGA Microspheres in the Alleviation of Neuropathic Pain via the CCI Model
Source: Front Pharmacol. 2022 Jun 13;13:910885. doi: 10.3389/fphar.2022.910885 (PMC9234216; doi:10.3389/fphar.2022.910885)

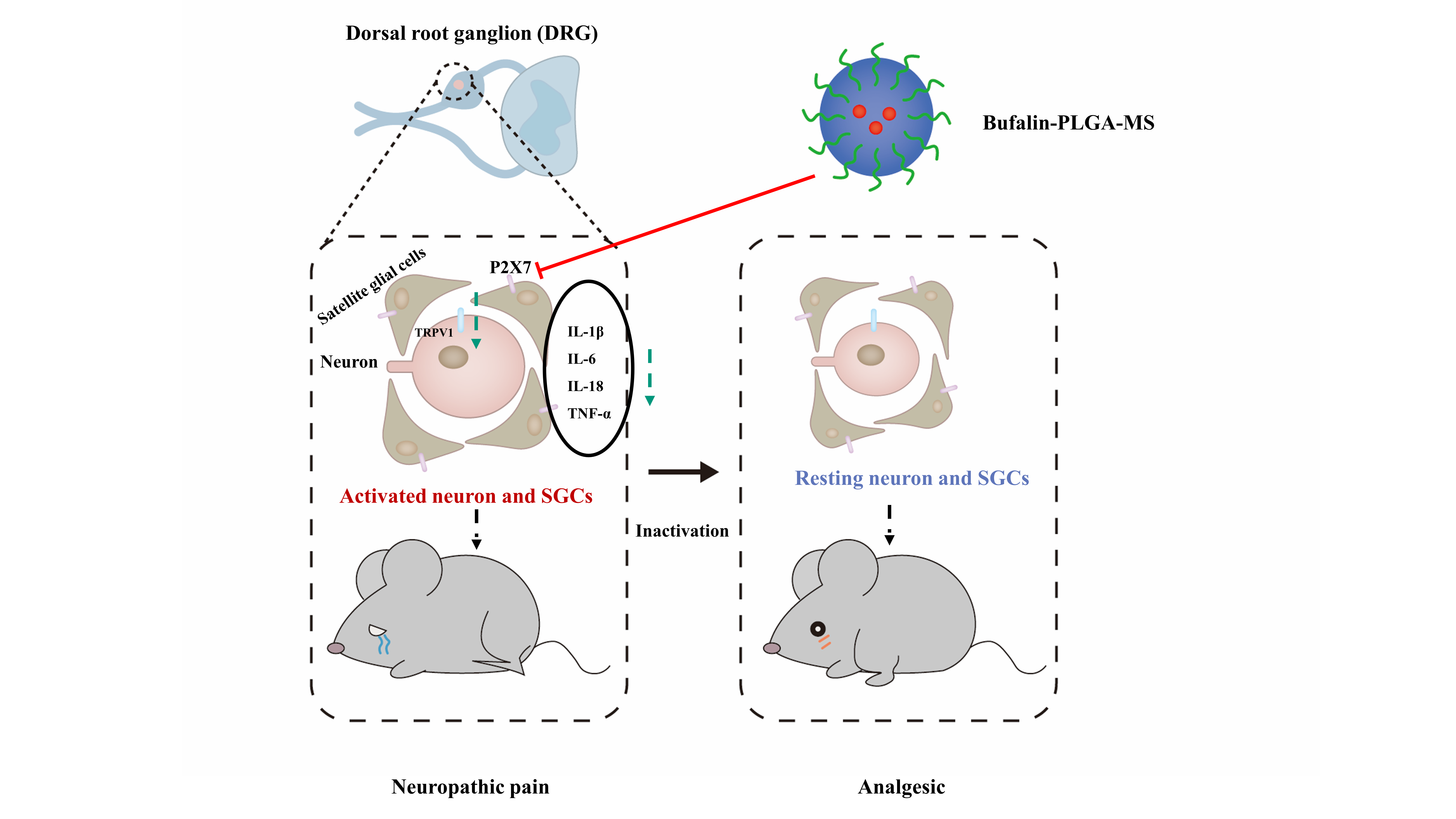

Supplement: Supplementary file 1 [file Image1.TIF]
